# Cytotoxic T-cell deficiency and sustained B-cell activation in serofast syphilis: a prospective observational study

**DOI:** 10.3389/fimmu.2026.1792505

**Published:** 2026-03-27

**Authors:** Konrad Kaminiów, Martyna Kiołbasa, Katarzyna Napiórkowska-Baran, Maciej Pastuszczak

**Affiliations:** 1 Clinical Department of Dermatology in Zabrze, Medical University of Silesia, Katowice, Poland; 2 Department of Allergology, Clinical Immunology and Internal Diseases, Collegium Medicum Bydgoszcz, Nicolaus Copernicus University Toruń, Toruń, Poland

**Keywords:** adaptive immunity, anticardiolipin antibodies, B-cell activation, cytotoxic T cells, host–pathogen interaction, immune dysregulation, *Treponema pallidum*

## Abstract

**Introduction:**

The serofast state, defined by persistent non-treponemal reactivity after adequate therapy for syphilis, remains a clinically relevant yet poorly understood condition. Whether serofast reflects residual infection or persistent immune activation remains uncertain. We aimed to characterize peripheral blood lymphocyte subpopulations and anticardiolipin antibodies in patients with serofast syphilis and compare them with individuals achieving a serological cure.

**Methods:**

We conducted a prospective observational study including adults with early syphilis treated with benzathine penicillin G. Peripheral blood samples were collected at baseline and six months post-treatment for immunophenotyping and antiphospholipid antibody assessment. Serological outcome was defined by Venereal Disease Research Laboratory (VDRL) response (serofast vs. serologically cured). Twelve healthy volunteers served as controls for anticardiolipin antibody testing.

**Results:**

Among 41 included patients, 9 (22%) developed a serofast state. Serofast patients demonstrated significantly lower baseline numbers of cytotoxic T lymphocytes (CD8^+^ and CD3^+^) compared with serologically cured individuals, a difference that persisted after treatment (all p<0.05). Six months post-treatment, serofast patients showed higher B-cell counts (p<0.05). Low-level anticardiolipin IgG and IgM were detectable in nearly all patients before treatment but had become undetectable in all participants at six months, while all healthy controls tested negative. No thrombotic events were observed.

**Discussion:**

Serofast syphilis was associated with reduced cytotoxic T-cell counts and persistent B-cell activation, supporting a model in which impaired cytotoxic clearance of antigen-presenting cells may prolong antibody production despite microbiological cure. The transient nature of infection-induced anticardiolipin antibodies further argues against persistent autoimmunity. These findings provide mechanistic insight into serofast responses and suggest potential immunological determinants of post-treatment serological persistence.

## 1 Introduction

Syphilis remains a major global sexually transmitted infection despite the availability of effective antimicrobial therapy. Current international guidelines consistently recommend long-acting benzathine penicillin G as the first-line and gold-standard treatment for all stages of syphilis, including early disease (1, 2). Treatment success is typically defined by clinical resolution of symptoms together with a serological response, characterized as at least a fourfold (two-dilution) decline in non-treponemal test titers such as VDRL or RPR within 6–12 months following therapy for early syphilis (3, 4).

Despite the absence of confirmed penicillin resistance in *Treponema pallidum*, numerous cohort studies and a systematic review have shown that many of appropriately treated patients fail to reach this expected serological decline (5–8). This so-called “serofast” state - persistent low non-treponemal titers not meeting criteria for either serological cure or treatment failure - has been observed in approximately 20–30% of patients, particularly in certain clinical or epidemiological subgroups (3, 5). The mechanisms underlying serofast remain unclear. It is still debated whether persistent titers reflect the continued presence of viable organisms or a dysregulated, prolonged host immune response with ongoing antibody production in the absence of active infection (9–11). This uncertainty is clinically relevant, as current guidelines provide limited and sometimes inconsistent recommendations regarding the management of patients who remain serofast after standard therapy (12). Another immunological feature historically associated with syphilis is the presence of antiphospholipid antibodies, particularly anticardiolipin antibodies (aCL). Infection-related aCL are generally transient, often β2-glycoprotein I - independent, and usually non-thrombogenic, distinguishing them from the persistent, β2-glycoprotein I - dependent antibodies characteristic of antiphospholipid syndrome (13, 14). However, contemporary data on the dynamics of aCL in early syphilis after penicillin therapy, and their relationship to serological treatment outcomes, remain limited.

Few studies have examined peripheral blood lymphocyte subsets in patients with serofast syphilis, and their findings have been inconsistent. Some reports describe alterations in T- and B-cell distributions or shifts toward enhanced humoral activity, whereas others do not confirm reproducible differences (15, 16). Emerging molecular data, including miRNA-based analyses, further suggest potential immune dysregulation, although these observations remain preliminary and are derived from small patient cohorts (17). Recent studies have also begun to explore the role of innate and cellular immune responses, including natural killer cell activity and T-cell–mediated pathways, in shaping serological outcomes in syphilis, although the available data remain limited and sometimes conflicting (9, 16, 18). Overall, available evidence is limited and inconclusive, underscoring the need for more comprehensive immunological characterization of serofast patients (5).

In this study, we aimed to address these gaps by performing a detailed longitudinal analysis of peripheral blood lymphocyte subpopulations and anticardiolipin antibodies in patients with early syphilis, assessed both before and after standard penicillin therapy. We compared individuals who developed a serofast state with those who achieved serological cure to identify immune correlates of serofast and to characterize the dynamics of aCL following treatment in this context.

## 2 Materials and methods

### 2.1 Study design

We performed a prospective observational study between 2024 and 2025 at the Clinical Department of Dermatology in Zabrze, Medical University of Silesia (Poland). All consecutive adults with newly diagnosed early syphilis who met inclusion/exclusion criteria were invited to participate. All consecutive eligible patients presenting during the study period were included. Because the study was designed as an exploratory prospective analysis, no formal *a priori* sample size calculation was performed. In addition, a convenience sample of healthy volunteers without a history of syphilis or autoimmune disease and with negative treponemal and non-treponemal tests was included as a control group for antiphospholipid antibody assessment. The healthy control group was included solely for reference measurements of antiphospholipid antibodies. No additional selection procedures applied. The study protocol was approved by the institutional ethics committee (BNW/NWN/0052/KB1/21/I/24), and written informed consent was obtained from each participant.

### 2.2 Eligibility criteria and clinical assessment

Participants were eligible if ≥18 years old with early syphilis (secondary or early-latent stage), based on CDC criteria (clinical presentation, exposure history, serology). Early latent syphilis was diagnosed when both treponemal and non-treponemal tests were reactive in the absence of clinical signs, provided that there was evidence of acquisition within the preceding 12 months, defined as (i) documented seroconversion after previously negative serologic tests, (ii) a sustained fourfold increase in non-treponemal titers, (iii) clinical symptoms consistent with primary or secondary syphilis during the past year, or (iv) a recent sexual exposure considered to be of high risk.

Exclusion criteria comprised: previous antimicrobial or immunosuppressive therapy within 6 months, HIV infection, chronic inflammatory or autoimmune disorders, and current anticoagulant/antiplatelet use (≥6 weeks before enrollment).

All patients received benzathine penicillin G 2.4 million units intramuscularly, administered at our clinical center under direct medical supervision in accordance with current guidelines, ensuring full treatment adherence. Baseline demographic and clinical data (age, sex, disease stage) were recorded at study entry.

### 2.3 Definition of serological outcome

Serological response was evaluated 6 months after treatment, corresponding to the time point of repeat immunological assessment. Patients were classified into two categories based on VDRL titer change (**Table 1**): (1) serofast – less than a fourfold decline in VDRL titer compared with baseline; and (2) serologically cured – at least a fourfold decline in VDRL titer. This definition follows criteria proposed by Sena et al. (5). Patients classified as serofast could exhibit either stable titers or minor fluctuations that did not reach the fourfold decline required to define serological cure. Clinical and serological follow-up continued up to 12 months, during which non-treponemal titers in serofast patients remained stable.

**Table 1:** Baseline and posttreatment clinical and laboratory characteristics of serofast and non-serofast patients.

Clinical and laboratory characteristics	Serofast group; n=9	Non-serofast group; n=32	p value
Females; n (%)	2 (22.2)	7 (21.9)	1.0
Age; years	31.5 (28.0-45.0)	34.0 (22.0-69.0)	0.2
secondary syphilis; n (%)	6 (66.7)	20 (62.5)	0.91
early latent syphilis; n (%)	3 (33.3)	12 (37.5)	0.87
baseline VDRL	64 (8-128)	64 (2-256)	0.44
baseline Hb concentration; d/dL	14.1 (12.7-16.7)	14.7 (11.6-17.9)	0.97
baseline erythrocytes count; x10^6^/uL	5.08 (4.4-5.4)	5.04 (3.8-5.6)	0.98
baseline leukocytes count; x10^3^/uL	6.7 (4.2-10.7)	7.0 (3.8-9.0)	0.74
baseline lymphocytes count; x10^3^/uL	1.3 (0.6-1.8)	1.6 (0.9-2.8)	0.04
baseline neutrophils count; x10^3^/uL	5.1 (2.1-8.1)	4.5 (2.1-6.8)	0.78
baseline eosinophils count; x10^3^/uL	0.08 (0.01-0.2)	0.09 (0.03-0.5)	0.67
baseline monocytes count; x10^3^/uL	0.5 (0.3-1.1)	0.56 (0.2-0.9)	0.89
baseline basophils count; x10^3^/uL	0.02 (0.01-0.03)	0.03 (0.01-0.06)	0.04
baseline platelets count; x10^3^/uL	260.5 (207.0-340.0)	259.0 (149.0-460.0)	0.77
baseline CRP levels; mg/dL	2.5 (1.3-33.0)	6.2 (0.00-23.4)	0.58
baseline C3 levels; mg/dL	1.3 (1.1-1.5)	1.3 (0.9-1.6)	0.85
baseline C4 levels; mg/dL	0.2 (0.1-0.35)	0.2 (0.1-0.5)	0.8
posttreatment VDRL	64 (8-128)	4.00 (0.00-16.00)	0.00004
posttreatment Hb concentration; d/dL	14.7 (12.9-17.2)	15.3 (13.0-17.5)	0.85
posttreatment erythrocytes count; x10^6^/uL	5.07 (4.5-5.7)	5.02 (4.1-5.8)	0.79
posttreatment leukocytes count; x10^3^/uL	7.2 (4.1-11.0)	6.2 (3.5-8.5)	0.13
posttreatment lymphocytes count; x10^3^/uL	1.95 (1.6-2.2)	1.9 (1.2-3.1)	0.82
posttreatment neutrophils count; x10^3^/uL	4.3 (1.7-7.9)	3.5 (1.9-5.7)	0.22
posttreatment eosinophils count; x10^3^/uL	0.17 (0.12-0.27)	0.1 (0.03-0.4)	0.05
posttreatment monocytes count; x10^3^/uL	0.75 (0.53-1.21)	0.52 (0.21-0.73)	0.007
posttreatment basophils count; x10^3^/uL	0.02 (0.01-0.03)	0.03 (0.01-0.06)	0.04
posttreatment platelets count; x10^3^/uL	267.0 (210.0-323.0)	248.0 (137.0-350.0)	0.56
posttreatment CRP levels; mg/dL	1.95 (0.00-3.5)	0.98 (0.00-22.6)	1.0
posttreatment C3 levels; mg/dL	1.1 (0.96-1.4)	1.2 (0.92-1.42)	0.95
posttreatment C4 levels; mg/dL	0.28 (0.14-0.29)	0.2 (0.09-0.4)	0.27

Data are given as median (min-max) or otherwise stated.

VDRL, Venereal Disease Research Laboratory test; Hb, Hemoglobin; CRP, C-reactive protein.

### 2.4 Blood sampling

Peripheral venous blood was collected at two time-points: before treatment (baseline) and six months after completion of therapy. Blood for serological and immunological analyses drawn into 3.2% sodium citrate tubes (9:1 blood-to-anticoagulant). Platelet-poor plasma prepared by double centrifugation (<10×10^9^/L) and stored at −80 °C if immediate analysis was not possible. In healthy controls, aCL IgG and IgM were measured using the same sampling procedure and laboratory assays.

### 2.5 Immunological assays

#### 2.5.1 Antiphospholipid antibodies

IgG and IgM anticardiolipin (aCL) and anti-β_2_-glycoprotein I (anti-β_2_GPI) antibodies were quantified using commercially available ELISAs (QUANTA Lite^®^, Inova Diagnostics/Diasorin). Results were expressed in GPL, MPL or standardized SGU/SMU units. Positivity was defined as ≥40 GPL/MPL or above the 99th percentile according to international criteria. Antibody measurements were performed at baseline and 6 months post-treatment.

#### 2.5.2 Lymphocyte subpopulation analysis

Peripheral blood lymphocyte subsets were determined in EDTA-anticoagulated whole blood using multicolor flow cytometry (e.g., FACSCanto II, BD Biosciences). The following monoclonal antibody panels were used for identification: T cells (CD3+), CD4+ helper T cells (CD3+CD4+), CD8+ cytotoxic T cells (CD3+CD8+), double-positive T cells (CD3+CD4+CD8+), B cells (CD19+), NK cells (CD3-CD56+), NKT cells (CD3+CD56+).

Absolute cell counts were obtained by dual-platform analysis using simultaneous complete blood count measurements. Data were processed using dedicated flow cytometry software (FACSDiva).

### 2.6 Syphilis serology

Non-treponemal testing was carried out with the VDRL assay (BD Macro-Vue™), and titers were determined by serial twofold dilutions. Treponema pallidum hemagglutination assay (TPHA, Serodia^®^, Fujirebio/Bio-Rad) was performed according to the manufacturer’s instructions. Only patients with concordant reactivity in both assays were included.

### 2.7 Statistical analysis

Continuous variables are presented as medians and minimum–maximum values, whereas categorical variables are expressed as counts and percentages. Between-group comparisons (serofast vs. serologically cured) were performed using the Mann–Whitney U test for continuous variables and the χ² test (or Fisher’s exact test, where appropriate) for categorical variables. Pre/post-treatment values were compared using paired non-parametric tests if applicable. A two-sided p value <0.05 was considered statistically significant. All statistical analyses were conducted using Statistica, version 13.3 (TIBCO Software Inc., Palo Alto, CA, USA).

## 3 Results

### 3.1 Study population

A total of 41 patients with early syphilis were included, of whom 9 (22%) developed a serofast state and 32 (78%) achieved a serological cure. Twelve healthy controls were additionally examined for antiphospholipid antibody assessment, all of whom had negative treponemal and non-treponemal tests. Baseline demographic and clinical characteristics, including age, sex and disease stage, were comparable between the serofast and non-serofast groups (**Table 1**). No significant differences were observed in baseline VDRL titers, hemoglobin concentration, erythrocyte count, platelet count, or complement components C3 and C4 (all p>0.05). Baseline leukocyte subpopulations were generally similar, except for lower lymphocyte and basophil counts in the serofast group (p=0.04 and p=0.04, respectively).

### 3.2 Posttreatment hematological and biochemical parameters

At six months, most clinical and routine laboratory parameters remained comparable between groups (**Table 1**). Patients with serofast demonstrated higher monocyte counts (p=0.007) and lower basophil counts (p=0.04) compared with non-serofast individuals, whereas differences in other leukocyte subsets were not statistically significant.

### 3.3 Lymphocyte subpopulations

**Figure 1:**
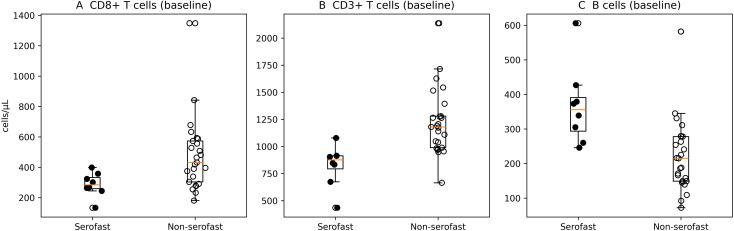
Baseline lymphocyte subsets in serofast and serologically cured patients.Box plots with individual data points illustrating baseline counts of **(A)** CD8^+^ T cells, **(B)** CD3^+^ T cells, and **(C)** B cells in patients who subsequently developed a serofast state and those who achieved serological cure. Boxes represent the interquartile range, horizontal lines indicate the median, filled circles represent serofast patients, and open circles represent non-serofast patients.

At baseline, patients who subsequently developed a serofast state exhibited lower absolute counts of CD3^+^, CD4^+^ and CD8^+^ T cells compared with the non-serofast group (p=0.00009, p=0.02 and p=0.01, respectively) (**Table 2**). Baseline B-cell counts were conversely higher among serofast patients (p=0.002). These differences in key lymphocyte subsets are illustrated in **Figure 1**.

**Table 2:** Baseline and posttreatment immunological parameters in serofast and non-serofast patients.

Parameters	Serofast group; n=9	Non-serofast group; n=32	p-value
Age; years	31.5 (28.0-45.0)	34.0 (22.0-69.0)	0.2
baseline CD3+ T cells/uL	876.0 (435.0-1079.0)	1180.0 (665.0-2137.0)	0.00009
baseline CD4+ T cells/uL	430.5 (213.0-726.0)	612.0 (289.0-1173.0)	0.02
baseline CD8+ T cells/uL	285.0 (134.0-399.0)	432.0 (181.0-1348.0)	0.01
baseline CD4+CD8+ T cells/uL	0.40 (0.30-0.50)	0.50 (0.20-1.30)	0.04
baseline CD4/CD8 ratio	1.75 (0.50-2.80)	1.40 (0.20-4.10)	0.89
baseline NK cells/uL	158.0 (51.0-592.0)	173.5 (83.0-465.0)	0.61
baseline NKT cells/uL	5.9 (2.8-16.0)	3.55 (0.9-14.5)	0.08
baseline B cells/uL	356.0 (246.0-606.0)	215.0 (72.0-582.0)	0.002
baseline aCL IgM; U/mL	22.86 (0.00-67.51)	10.19 (0.00-120.00)	0.95
baseline aCL IgG; U/mL	17.48 (0.00-61.66)	11.46 (0.00-120.00)	0.92
baseline anti-β2GPI; U/mL	2.61 (2-4.2)	2.58 (2-3.8)	0.89
posttreatment CD3+ T cells/uL	1143.0 (984.0-1594.0)	1296.0 (826.0-3066.0)	0.49
posttreatment CD4+ T cells/uL	582.0 (465.0-1081.0)	680.0 (395.0-1924.0)	0.61
posttreatment CD8+ T; cells/uL	347.0 (290.0-582.0)	432.0 (215.0-1554.0)	0.49
posttreatment CD4+CD8+ T cells/uL	0.4 (0.2-0.4)	0.4 (0.4-0.7)	0.02
posttreatment CD4/CD8 ratio	1.6 (1.0-2.8)	1.5 (0.4-4.0)	0.54
posttreatment NK cells/uL	403.0 (175.0-745.0)	236.0 (139.0-630.0)	0.15
posttreatment NKT cells/uL	4.8 (3.1-6.5)	2.7 (0.2-17.4)	0.12
posttreatment B cells/uL	380.0 (197.0-497.0)	235.0 (118.0-584.0)	0.01
posttreatment aCL IgM; U/mL	0.00 (0.00-6.72)	0.00 (0.00-14.00)	0.98
posttreatment aCL IgG; U/mL	0.00 (0.00-6.16)	0.00 (0.00-5.37)	0.67
posttreatment anti-β2GPI; U/mL	2.78 (2-3.7)	2.58 (2-4.1)	0.83

Data are given as median (min-max) or otherwise stated.

NK cells, Natural killer cells; NKT cells, Natural killer T cells; aCL, Anticardiolipin antibodies; anti-β2GPI, Anti–beta-2-glycoprotein I antibodies.

Baseline double-positive CD4^+^CD8^+^ T cells were also reduced in the serofast group (p=0.04), whereas NKT cells showed a non-significant trend towards higher values (p=0.08).

After treatment, most lymphocyte subsets no longer differed between groups (all p>0.05), except for CD4^+^CD8^+^ T cells, which remained lower in the serofast group (p=0.02), and B cells, which were higher compared with non-serofast patients (p=0.01).

### 3.4 Anticardiolipin antibodies

**Figure 2:**
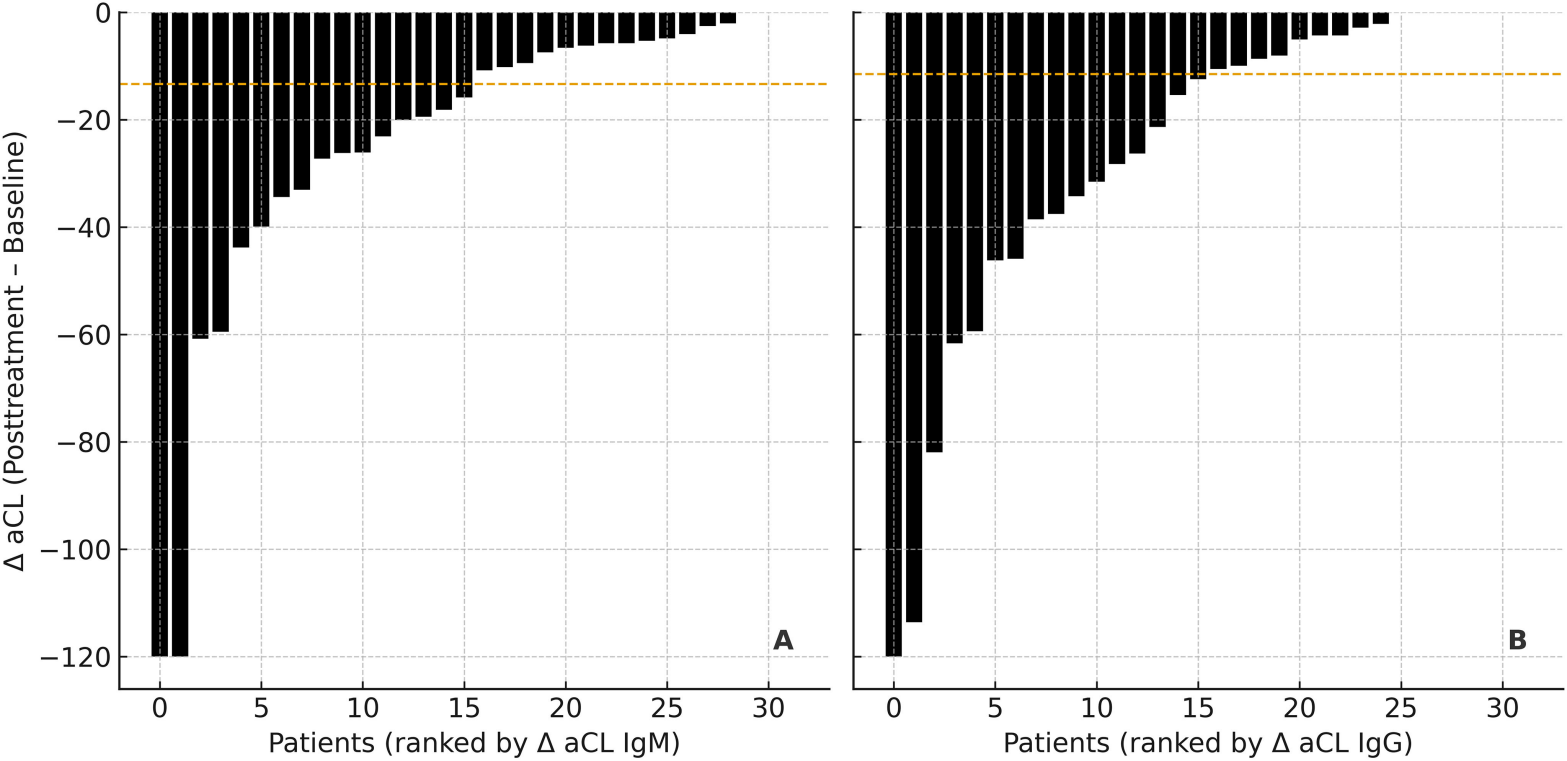
Waterfall plots demonstrating changes in anticardiolipin (aCL) antibody levels following treatment for early syphilis. **(A)** Δ aCL IgM (posttreatment – baseline) and **(B)** Δ aCL IgG (posttreatment – baseline) values are shown for each patient, ranked from the largest to the smallest decline. Each bar represents an individual participant. Negative values indicate a reduction in antibody titers after treatment. Dotted horizontal lines denote median changes for the cohort.

At baseline, low-level aCL IgM and IgG were detectable in most of patients in both groups, although median titers remained below the laboratory cut-off for positivity. By contrast, aCL IgG and IgM were undetectable in all 12 healthy controls. There were no significant differences between serofast and non-serofast patients (p = 0.95 and p = 0.92, respectively). Anti-β_2_GPI concentrations were likewise low and comparable between groups (p = 0.89) (**Table 2**). After treatment, aCL IgM and IgG titers showed a pronounced decline and became undetectable in almost all patients, again with no differences between serofast and non-serofast individuals (p = 0.98 and p = 0.67, respectively). Posttreatment anti-β_2_GPI levels remained similar across groups (p = 0.83) (**Table 2**). The uniform decrease in aCL titers is illustrated by the waterfall plots (**Figure 2**), where virtually all patients demonstrated a negative Δ (posttreatment – baseline), with median changes below zero in both IgM and IgG panels.

## 4 Discussion

The serofast state following adequate treatment for early syphilis is a long-recognized yet unresolved clinical issue. Despite clinical cure and clinical resolution, up to 20–30% of patients do not achieve a fourfold decline in non-treponemal antibody titers within the expected 6–12 months, creating uncertainty regarding persistent infection versus sustained humoral activation. Recent reports highlight that the mechanisms underlying serofast remain insufficiently understood and may differ between individuals (3, 19). Importantly, extending penicillin treatment does not consistently improve the serological response in serofast patients, suggesting that persistent non-treponemal reactivity is unlikely to reflect ongoing bacterial replication, but rather represents a state of prolonged antibody production despite pathogen clearance (20, 21).

In the present study, we provide new evidence indicating that individuals who subsequently developed a serofast state displayed, already at baseline, significantly lower numbers of cytotoxic T lymphocytes (CD8^+^ as well as CD3^+^) compared with those who achieved conventional serological cure. This deficiency persisted six months after treatment. These findings support the hypothesis that the ability to mount an adequate cytotoxic T-cell response early during infection may influence subsequent serological evolution. To our knowledge, this is the first prospective study linking quantitative cytotoxic T-cell disturbance with serofast outcome.

From an immunological perspective, cytotoxic T lymphocytes constitute a major effector arm of cell-mediated immunity and are crucial for controlling intracellular pathogens through cytolytic mechanisms and cytokine release, notably IFN-γ and TNF-α, which activate macrophages (10, 22, 23). Although *Treponema pallidum* is generally regarded as an extracellular organism, several studies suggest that partial intracellular residence, antigen uptake by professional antigen-presenting cells, and cross-presentation on MHC class I may engage cytotoxic pathways (23–25). In this context, reduced cytotoxic T-cell availability or responsiveness could impair efficient elimination of antigen-presenting cells (APCs) loaded with treponemal antigens, allowing prolonged antigen presentation to B cells. In clinical terms, these observations raise the possibility that, in the absence of antibiotic therapy, individuals with poorer cytotoxic T-cell responses might struggle to clear the pathogen spontaneously.

In our cohort, the higher B-cell counts observed six months after therapy in serofast individuals further support a scenario of prolonged antigen-driven stimulation. Given that penicillin effectively eradicates viable *T. pallidum*, sustained non-treponemal antibodies in serofast patients are more likely attributable to ongoing immune activation rather than occult infection. One mechanistic explanation is that incomplete clearance of APCs presenting treponemal antigens could maintain B-cell activation and antibody secretion despite microbiological cure. Although this interpretation remains speculative and was not directly tested in our study, it is consistent with current concepts linking cytotoxic T-cell function with regulation of antigen presentation and downstream B-cell activation. This concept places cytotoxic T-cell competence as a potential immunological determinant of serofast.

The detection of antiphospholipid antibodies (aPL), particularly anticardiolipin antibodies (aCL), provides an additional window into immune activation in syphilis. aPL constitute a heterogeneous family of autoantibodies directed against phospholipids or phospholipid-binding proteins, and their persistent presence - especially anti-β_2_-glycoprotein I - is pathognomonic for the antiphospholipid syndrome (APS), a prothrombotic autoimmune disorder (26). Infections, however, are well-documented triggers of transient aPL responses, including in syphilis, viral hepatitis, HIV, tuberculosis, and others (21, 27, 28). Importantly, in syphilis the antibodies are typically β_2_GPI-independent, bind “naked” cardiolipin, and do not confer a significant thrombotic risk (21, 22). Indeed, wide variation has been reported in the prevalence of aCL during syphilis (approximately 6–70%), depending on stage and assay methodology (20, 28).

Consistent with previous observations, our study demonstrates that low-level aCL IgG and IgM were detectable in virtually all patients before therapy, whereas none remained detectable six months after treatment. The complete disappearance of aCL following penicillin therapy is in line with an infection-driven phenomenon and argues against persistent autoimmunity. Of note, all healthy controls tested negative for both aCL isotypes, supporting the concept that the aCL detected in our patients were induced by infection and resolved with pathogen clearance.

Collectively, our findings support a conceptual model in which poorer cytotoxic T-cell reserves may be associated with delayed removal of APCs presenting treponemal antigens, leading to prolonged antigen-driven B-cell activation and sustained antibody production. This immunological environment could foster persistent non-treponemal titers in the absence of active infection. While such a model does not exclude other mechanisms, it provides a biologically plausible explanation that reconciles microbiological cure with persistent serological reactivity.

The strengths of our study include its prospective design, standardized therapy, longitudinal immunophenotyping before and after treatment, and the inclusion of a healthy control group to contextualize aCL dynamics. Furthermore, the simultaneous evaluation of cytotoxic T-cell and B-cell subsets provides complementary mechanistic insights. However, several limitations should be acknowledged. The relatively small sample size should be considered when interpreting the findings. In addition, the study evaluated quantitative lymphocyte subsets rather than functional cytotoxic T-cell activity (e.g., degranulation assays or cytokine profiling), and therefore mechanistic interpretations should be viewed as hypothesis-generating. Moreover, while lower CD8^+^ numbers may indicate diminished cytotoxic potential, we cannot exclude contributions from other immune compartments such as regulatory T cells, NK cells, or monocyte subsets.

Future research should seek to confirm our findings in larger cohorts and incorporate functional immunological assessments. Longitudinal studies examining memory B-cell dynamics, germinal center reactions, and APC phenotype may further clarify the mechanisms driving persistent antibody production after treatment. Additionally, the role of T-cell exhaustion, senescence, or checkpoint pathways warrants investigation, particularly in patients demonstrating delayed serological responses.

From a clinical perspective, understanding whether serofast reflects immunological dysregulation rather than microbiological persistence has important implications. If confirmed, this paradigm would discourage unnecessary additional antibiotic courses and shift focus toward biomarkers of immune clearance and recovery. Carefully designed immunophenotyping may identify patients at risk for serofast and guide tailored follow-up strategies. Ultimately, integrating immunological profiling with clinical parameters could refine the management of syphilis beyond traditional serological endpoints.

In summary, this study provides novel evidence linking baseline cytotoxic T-cell deficits and persistent B-cell activation with the development of serofast responses in early syphilis, alongside complete normalization of infection-induced aCL following treatment. These findings support a model in which impaired cytotoxic clearance of antigen-presenting cells may contribute to prolonged humoral stimulation despite microbiological cure. Future studies integrating functional T-cell assays and longitudinal immunophenotyping will be necessary to determine whether cytotoxic T-cell competence represents a true determinant of serological resolution in syphilis.

## Data Availability

The original contributions presented in the study are included in the article/supplementary material. Further inquiries can be directed to the corresponding author.
